# Ps and Qs: Quantization-Aware Pruning for Efficient Low Latency Neural Network Inference

**DOI:** 10.3389/frai.2021.676564

**Published:** 2021-07-09

**Authors:** Benjamin Hawks, Javier Duarte, Nicholas J. Fraser, Alessandro Pappalardo, Nhan Tran, Yaman Umuroglu

**Affiliations:** ^1^ Fermi National Accelerator Laboratory, Batavia, IL, United States; ^2^ University of California San Diego, La Jolla, CA, United States; ^3^ Xilinx Research, Dublin, Ireland; ^4^ Northwestern University, Evanston, IL, United States

**Keywords:** pruning, quantization, neural networks, generalizability, regularization, batch normalization

## Abstract

Efficient machine learning implementations optimized for inference in hardware have wide-ranging benefits, depending on the application, from lower inference latency to higher data throughput and reduced energy consumption. Two popular techniques for reducing computation in neural networks are pruning, removing insignificant synapses, and quantization, reducing the precision of the calculations. In this work, we explore the interplay between pruning and quantization during the training of neural networks for ultra low latency applications targeting high energy physics use cases. Techniques developed for this study have potential applications across many other domains. We study various configurations of pruning during quantization-aware training, which we term *quantization-aware pruning*, and the effect of techniques like regularization, batch normalization, and different pruning schemes on performance, computational complexity, and information content metrics. We find that quantization-aware pruning yields more computationally efficient models than either pruning or quantization alone for our task. Further, quantization-aware pruning typically performs similar to or better in terms of computational efficiency compared to other neural architecture search techniques like Bayesian optimization. Surprisingly, while networks with different training configurations can have similar performance for the benchmark application, the information content in the network can vary significantly, affecting its generalizability.

## 1 Introduction

Efficient implementations of machine learning (ML) algorithms provide a number of advantages for data processing both on edge devices and at massive data centers. These include reducing the latency of neural network (NN) inference, increasing the throughput, and reducing power consumption or other hardware resources like memory. During the ML algorithm design stage, the computational burden of NN inference can be reduced by eliminating nonessential calculations through a modified training procedure. In this paper, we study efficient NN design for an ultra-low latency, resource-constrained particle physics application. The classification task is to identify radiation patterns that arise from different elementary particles at sub-microsecond latency. While our application domain emphasizes low latency, the generic techniques we develop are broadly applicable.

Two popular techniques for efficient ML algorithm design are *quantization* and *pruning*. Quantization is the reduction of the bit precision at which calculations are performed in a NN to reduce the memory and computational complexity. Often, quantization employs fixed-point or integer calculations, as opposed to floating-point ones, to further reduce computations at no loss in performance. Pruning is the removal of unimportant weights, quantified in some way, from the NN. In the most general approach, computations are removed, or pruned, one-by-one from the network, often using their magnitude as a proxy for their importance. This is referred to as magnitude-based unstructured pruning, and in this study, we generically refer to it as pruning. Recently, quantization-aware training (QAT), accounting for the bit precision at training time, has been demonstrated in a number of studies to be very powerful in efficient ML algorithm design. In this paper, we explore the potential of combining pruning with QAT at any possible precision. As one of the first studies examining this relationship, we term the combination of approaches *quantization-aware pruning (QAP)*. The goal is to understand the extent to which pruning and quantization approaches are complementary and can be optimally combined to create even more efficiently designed NNs.

Furthermore, as detailed in Section. 1.1, there are multiple approaches to efficient NN optimization and thus also to QAP. While different approaches may achieve efficient network implementations with similar classification performance, these trained NNs may differ in their information content and computational complexity, as quantified through a variety of metrics. Thus, some approaches may better achieve other desirable characteristics beyond classification performance such as algorithm robustness or generalizability.

This paper is structured as follows. Section 1.1 briefly recapitulates related work. Section 2 describes the low latency benchmark task in this work related to jet classification at the CERN Large Hadron Collider (LHC). Section 3 introduces our approach to QAP and the various configurations we explore in this work. To study the joint effects of pruning and quantization, we introduce the metrics we use in Section 4. The main results are reported in Section 5. Finally, a summary and outlook are given in Section 6.

### 1.1 Related Work

While NNs offer tremendous accuracy on a variety of tasks, they typically incur a high computational cost. For tasks with stringent latency and throughput requirements, this necessitates a high degree of efficiency in the deployment of the NN. A variety of techniques have been proposed to explore the efficient processing of NNs, including quantization, pruning, low-rank tensor decompositions, lossless compression and efficient layer design. We refer the reader to Sze et al. (2020) for a survey of techniques for efficient processing of NNs, and focus on related work around the key techniques covered in this paper.

#### Pruning

Early work (LeCun et al., 1990) in NN pruning identified key benefits including better generalization, fewer training examples required, and improved speed of learning the benefits through removing insignificant weights based on second-derivative information. Recently, additional compression work has been developed in light of mobile and other low-power applications, often using magnitude-based pruning (Han et al., 2016). In Frankle and Carbin (2019), the authors propose the *lottery ticket (LT) hypothesis*, which posits that sparse subnetworks exist at initialization which train faster and perform better than the original counterparts. Renda et al. (2020) proposes learning rate rewinding in addition to weight rewinding to more efficiently find the winning lottery tickets. Zhou et al. (2019) extends these ideas further to learning “supermasks” that can be applied to an untrained, randomly initialized network to produce a model with performance far better than chance. The current state of pruning is reviewed in Blalock et al. (2020), which finds current metrics and benchmarks to be lacking.

#### Quantization

Reducing the precision of a static, trained network’s operations, *post-training quantization (PTQ)*, has been explored extensively in the literature (Han et al., 2016; Duarte et al., 2018; Banner et al., 2019; Meller et al., 2019; Nagel et al., 20192019; Zhao et al., 2019). QAT (Courbariaux et al., 2015; Rastegari et al., 2016a; Li and Liu, 2016; Zhou et al., 2016; Moons et al., 2017; Hubara et al., 2018; Micikevicius et al., 2018; Wang et al., 2018; Zhang et al., 2018; Zhuang et al., 2018; Ngadiuba et al., 2020) has also been suggested with different frameworks like QKeras (Coelho, 2019; Coelho et al., 2021) and Brevitas (Blott et al., 2018; Pappalardo, 2020) developed specifically to explore quantized NN training. Hessian-aware quantization (HAWQ) (Dong et al., 2019; Dong et al., 2020) is another quantization approach that uses second derivative information to automatically select the relative bit precision of each layer. The Bayesian bits approach attempts to unify structured pruning and quantization by identifying pruning as the 0-bit limit of quantization (van Baalen et al., 2020). In Hacene et al. (2020), a combination of a pruning technique and a quantization scheme that reduces the complexity and memory usage of convolutional layers, by replacing the convolutional operation by a low-cost multiplexer, is proposed. In partuclar, the authors propose an efficient hardware architecture implemented on field-programmable gate array (FPGA) on-chip memory. In Chang et al. (2021), the authors apply different quantization schemes (fixed-point and sum-power-of-two) to different rows of the weight matrix to achieve better utilization of heterogeneous FPGA hardware resources.

#### Efficiency Metrics

Multiple metrics have been proposed to quantify NN efficiency, often in the context of dedicated hardware implementations. The artificial intelligence quotient (aiQ) is proposed in Schaub and Hotaling (2020) as metric to measure the balance between performance and efficiency of NNs. Bit operations (BOPs) (Baskin et al., 2021) is another metric that aims to generalize floating-point operations (FLOPs) to heterogeneously quantized NNs. A hardware-aware complexity metric (HCM) (Karbachevsky et al., 2021) has also been proposed that aims to predict the impact of NN architectural decisions on the final hardware resources. Our work makes use of some of these metrics and further explores the connection and tradeoff between pruning and quantization.

## 2 Benchmark Task

The LHC is a proton-proton collider that collides bunches of protons at a rate of 40 MHz. To reduce the data rate, an online filter, called the trigger system, is required to identify the most interesting collisions and save them for offline analysis. A crucial task performed on FPGAs in the Level-1 trigger system that can be greatly improved by ML, both in terms of latency and accuracy, is the classification of particles coming from each proton-proton collision. The system constraints require algorithms that have a latency of O(*μ*s) while minimizing the limited FPGA resources available in the system.

**FIGURE 1:**
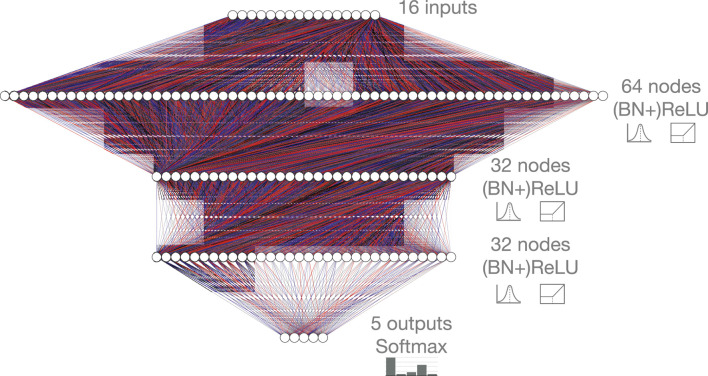
Baseline fully-connected neural network architecture, consisting of 16 inputs, five softmax-activated outputs, and three hidden layers. The three hidden layers contain 64, 32, and 32 hidden nodes each with ReLU activation. A configuration with batch normalization (BN) layers before each ReLU activation function is also considered. The red and blue lines represent positive and negative weights, respectively, and the opacity represents the magnitude of each weight for this randomly initialized network.

We consider a benchmark dataset for this task to demonstrate our proposed model efficiency optimization techniques. In Coleman et al. (2018), Duarte et al. (2018), and Moreno et al. (2020), a dataset (Pierini et al., 2020) was presented for the classification of collimated showers of particles, or *jets*, arising from the decay and hadronization of five different classes of particles: light flavor quarks (q), gluons (g), W and Z bosons, and top quarks (t). For each class, jets are pair-produced ($W^{+}W^{-},ZZ,q\bar{q},t\bar{t},gg$) in proton-proton collisions at a center-of-mass energy of 13 TeV from the same $q\bar{q}$ initial state. The jets are selected such that the unshowered parton or boson has a transverse momentum of 1 TeV within a narrow window of ±1%(10 GeV) such that transverse momenta spectra is similar for all classes. Each jet is represented by 16 physics-motivated high-level features which are presented in Table 1 of Coleman et al. (2018). The dataset contains 870,000 jets, balanced across all classes and split into 472,500 jets for training, 157,500 jets for validation, and 240,000 jets for testing. Adopting the same baseline architecture as in Duarte et al. (2018), we consider a fully-connected NN consisting of three hidden layers (64, 32, and 32 nodes, respectively) with rectified linear unit (ReLU) (Nair and Hinton, 2010; Glorot et al., 2011) activation functions, shown in Figure 1. The output layer has five nodes, yielding a probability for each of the five classes through a softmax activation function. We refer to this network as the baseline floating-point model.

**TABLE 1:** Performance evolution of the jet substructure classification task for this NN architecture.

Model	Precision	BN or *L_1_*	Pruned [%]	BOPs	Accuracy [%]	$\langle \epsilon_{b}^{\epsilon_{s}=0.5}\rangle$ [%]	〈AUC〉 [%]
Nominal	32-bit floating-point	*L*_1_ + BN	0	4,652,832	**76.977**	**0.00171**	**94.335**
Pruning + PTQ	16-bit fixed-point	*L*_1_ + BN	70	631,791	75.01	0.00210	94.229
QAT	6-bit fixed-point	*L*_1_ + BN	0	412,960	76.737	0.00208	94.206
QAP	6-bit scaled-integer	*L*_1_ + BN	**80**	**189,672**	76.602	0.00211	94.197

“Nominal” refers to an unpruned 32-bit implementation, “pruning + PTQ” refers to a network with FT pruning at 32-bit precision with PTQ applied to reduce the precision to 16 bits, “QAT” refers to a QKeras implementation, and “QAP” is this result. The bolded value in each column indicates the best value of each metric.

## 3 Quantization-Aware Pruning

Applying quantization and pruning to a NN can drastically improve its efficiency with little to no loss in performance. While applying these changes to a model post-training can be successful, to be maximally effective, we consider these effects at the time of NN training. Because computational complexity, as defined in Section 4, is quadratically dependent on precision while it is linearly dependent on pruning, the first step in our QAP approach is to perform QAT. This is followed by integrating pruning in the procedure.

### 3.1 Quantization-Aware Training

Quantized (Hubara et al., 2018; Gong et al., 2014; Wu et al., 2016; Vanhoucke et al., 2011; Gupta et al., 2015) and even binarized (Courbariaux et al., 2015; Gupta et al., 2015; Hubara et al., 2016; Rastegari et al., 2016b; Merolla et al., 2016) NNs have been studied as a way to compress NNs by reducing the number of bits required to represent each weight and activation value. As a common platform for NNs acceleration, FPGAs provide considerable freedom in the choice of data type and precision. Both choices should be considered carefully to prevent squandering FPGA resources and incurring additional latency. For example, in QKeras and hls4ml (Duarte et al., 2018), a tool for transpiling NNs on FPGAs, fixed-point arithmetic is used, which requires less resources and has a lower latency than floating-point arithmetic. For each parameter, input, and output, the number of bits used to represent the integer and fractional parts can be configured separately. The precision can be reduced through PTQ, where pre-trained model parameters are clipped or rounded to lower precision, without causing a loss in performance (Gupta et al., 2015) by carefully choosing the bit precision.

Compared to PTQ, a larger reduction in precision can be achieved through QAT (Li and Liu, 2016; Moons et al., 2017), where the reduced precision of the weights and biases are accounted for directly in the training of the NN. It has been found that QAT models can be more efficient than PTQ models while retaining the same performance (Coelho et al., 2021). In these studies, the same type of quantization is applied everywhere. More recently (Dong et al., 2019; Wang et al., 2019; Dong et al., 2020), it has been suggested that per-layer heterogeneous quantization is the optimal way to achieve high accuracy at low resource cost. For the particle physics task with a fully-connected NN, the accuracy of the reduced precision model is compared to the 32-bit floating-point implementation as the bit width is scanned. In the PTQ case (Duarte et al., 2018), the accuracy begins to drop below 14-bit fixed-point precision, while in the QAT case implemented with QKeras (Coelho et al., 2021) the accuracy is consistent down to 6 bits.

In this work, we take a different approach to training quantized NNs using Brevitas (Pappalardo, 2020), a PyTorch library for QAT. Brevitas provides building blocks at multiple levels of abstraction to compose and apply quantization primitives at training time. The goal of Brevitas is to model the data type restrictions imposed by a given target platform along the forward pass. Given a set of restriction, Brevitas provides several alternative learning strategies to fulfill them, which are exposed to the user as hyperparameters. Depending on the specifics of the topology and the overall training regimen, different learning strategies can be more or less successful at preserving the accuracy of the output NN. Currently, the available quantizers target variations of binary, ternary, and integer data types. Specifically, given a real valued input *x*, the integer quantizer $Q_{\text{int}}\left(x\right)$ performs uniform affine quantization, defined as

$$Q_{\text{int}}\left(x\right)=s\underset{y_{\text{min}},y_{\text{max}}}{\text{clamp}}\left(\text{round}\left(\frac{x}{s}\right)\right)$$ (1)

where

$$\underset{y_{\text{min}},y_{\text{max}}}{\text{clamp}}\left(y\right)=\{\begin{array}{ll} y_{\text{min}} & y<y_{\text{min}}\;,\\ y & y_{\text{min}}\leq y\leq y_{\text{max}}\;,\\ y_{\text{max}} & y>y_{\text{max}}\;, \end{array}$$ (2)

round(⋅):ℝ→ℤ is a rounding function, s∈ℝ is the *scale factor*, and $y_{\text{min}}\in \mathbb{Z}$ and $y_{\text{max}}\in \mathbb{Z}$ are the minimum and maximum thresholds, respectively, which depend on the available word length (number of bits in a word).

In this work, we adopt round-to-nearest as the round function, and perform per-tensor quantization on both weights and activations, meaning that *s* is constrained to be a scalar floating-point value. As the ReLU activation function is used throughout, unsigned values are used for quantized activations. Thus, for a word length of *n*, the clamp function, ${\text{champ}}_{A_{\text{min}},A_{\text{max}}}\left(\cdot \right)$, is used with $A_{\text{min}}=0$ and $A_{\text{max}}=2^{n}-1$. Quantized weights are constrained to symmetric signed values so ${\text{champ}}_{w_{\text{min}},w_{\text{max}}}\left(\cdot \right)$ is used with $w_{\text{max}}=2^{n-1}-1$ and $w_{\text{min}}=-w_{\text{max}}$.

In terms of learning strategies, we apply the straight-through estimator (STE) (Courbariaux et al., 2015) during the backward pass of the rounding function, which assumes that quantization acts as the identity function, as is typically done in QAT. For the weights’ scale, similar to Jacob et al. (2018), $s_{w}$ is re-computed at each training step such that the maximum value in each weight tensor is represented exactly

$$s_{w}=\frac{{\text{max}}_{\text{tensor}}\left(\left|W\right|\right)}{2^{n-1}-1}\;,$$ (3)

where W is the weight tensor for a given layer and ${\text{max}}_{\text{tensor}}\left(\cdot \right)$ is the function that takes an input tensor and returns the maximum scalar value found within. For the activations, the scale factor $s_{A}$ is defined as:

$$s_{A}=\frac{s_{A,\text{learned}}}{2^{n-1}}\;,$$ (4)

where $s_{A,\text{learned}}$ is a parameter individual to each quantized activation layer, initialized to 6.0 (in line with the ReLU6(⋅) activation function), and learned by backpropagation in logarithmic scale, as described in Jain et al. (2020). In the following, we refer to this scheme as scaled-integer quantization.

### 3.2 Integrating Pruning

Network compression is a common technique to reduce the size, energy consumption, and overtraining of deep NNs (Han et al., 2016). Several approaches have been successfully deployed to compress networks (Cheng et al., 2018; Choudhary et al., 2020; Deng et al., 2020). Here we focus specifically on *parameter pruning*: the selective removal of weights based on a particular ranking (Louizos et al., 2018; Frankle and Carbin, 2019; Blalock et al., 2020; Renda et al., 2020).

Prior studies (Duarte et al., 2018) have applied pruning in an iterative fashion: by first training a model then removing a fixed fraction of weights per layer then retraining the model, while masking the previously pruned weights. This processed can be repeated, restoring the final weights from the previous iteration, several times until reaching the desired level of compression. We refer to this method as fine-tuning (FT) pruning. While the above approach is effective, we describe here an alternative approach based on the LT hypothesis (Frankle and Carbin, 2019) where the remaining weights after each pruning step are initialized back to their original values (“weight rewinding”). We refer to this method as LT pruning. We propose a new hybrid method for constructing efficient NNs, QAP, which combines a pruning procedure with training that accounts for quantized weights. As a first demonstration, we use Brevitas (Pappalardo, 2020) to perform QAT and iteratively prune a fraction of the weights following the FT pruning method. In this case, we FT prune approximately 10% of the original network weights (about 400 weights) each iteration, with a reduction in the number of weights to prune once a sparsity of 90% is reached. Weights with the smallest $L_{1}$ norms across the full model are removed each iteration.

**FIGURE 2:**
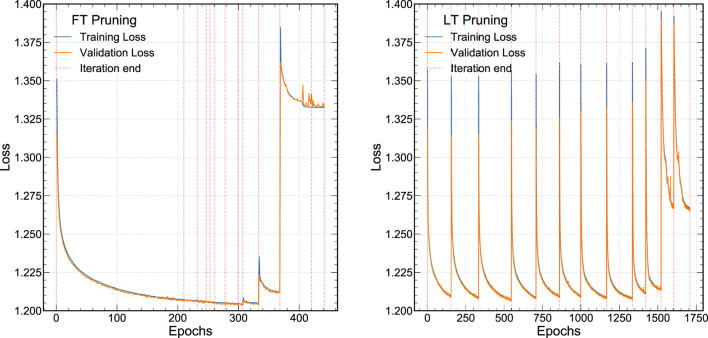
The loss function for the QAP procedure for a 6-bit jet classification neural network. FT pruning is demonstrated on the left **(A)** and LT pruning is shown on the right **(B)**.

Our procedure for FT and LT pruning are demonstrated in Figure 2, which shows the training and validation loss as a function of the epoch. To demonstrate the effect of QAP, we start by training a network using QAT for our jet substructure task constraining the precision of each layer to be 6 bits using Brevitas. This particular training includes batch normalization (BN) layers and $L_{1}$ regularization described in more detail in Section 3.3, although we also present results without these aspects.

In Figure 2A, the FT pruning procedure iteratively prunes the 6-bit weights from the network. Each iteration is denoted by the dotted red lines after which roughly 10% of the lowest magnitude weights are removed. At each iteration, we train for 250 epochs with an early stopping criteria of no improvement in the validation loss for 10 epochs. The FT pruning procedure continues to minimize or maintain the same loss over several pruning iterations until the network becomes so sparse that the performance degrades significantly around epoch 300. In Figure 2B, the LT pruning procedure is shown. Our approach deviates from the canonical LT pruning study (Frankle and Carbin, 2019) in that we fully train each pruning iteration until the early stopping criteria is satisfied instead of partially optimizing the network. This is because we would like to explore the performance of the network at each stage of pruning to evaluate a number of metrics. However, the behavior is as expected—at each pruning iteration the loss goes back to its initial value. Similar to the FT pruning case, when the LT pruning NN becomes very sparse, around epoch 1,500, the performance begins to degrade. We note that because of the additional introspection at each iteration, our LT pruning procedure requires many more epochs to train than the FT pruning procedure.

### 3.3 Neural Network Training Configurations

In this section, we describe BN and $L_{1}$ regularization, which have the power to modify the efficiency of our QAP models. We also describe Bayesian optimization (BO), which we use to perform a standard neural architecture search for comparison to QAP.

#### 3.3.1 Batch Normalization and $L_{1}$ Regularization

BN (Ioffe et al., 2015) was originally proposed to mitigate internal covariate shift, although others have suggested its true benefit is in improving the smoothness of the loss landscape (Santurkar et al., 2018). The BN transformation y for an input x is

$$y=\gamma \frac{x-\mu }{\sqrt{\sigma^{2}+\epsilon }}+\beta ,$$ (5)

given the running mean μ and standard deviation σ, the learnable scale γ and shift *β* parameters, and *ϵ* a small number to increase stability. Practically, the BN layer shifts the output of dense layers to the range of values in which the activation function is nonlinear, enhancing the network’s capability of modeling nonlinear responses, especially for low bit precision (Ngadiuba et al., 2020). For this reason, it is commonly used in conjunction with extremely low bit precision.

We also train models with and without $L_{1}$ regularization (Han et al., 2015; Duarte et al., 2018), in which the classification loss function $L_{\text{c}}$ is augmented with an additional term,

$$L=L_{\text{c}}+\lambda {\left|\left|w\right|\right|}_{1}\;,$$ (6)

where w is a vector of all the weights of the model and *λ* is a tunable hyperparameter. This can be used to assist or accelerate the process of iterative pruning, as it constrains some weights to be small, producing already sparse models (Ng, 2004). As the derivative of the penalty term is *λ* whose value is independent of the weight, $L_{1}$ regularization can be thought of as a force that subtracts some constant from an ineffective weight each update until the weight reaches zero.

#### 3.3.2 Bayesian Optimization

BO (Jones et al., 1998; O'Hagan, 1978; Osborne, 2010) is a sequential strategy for optimizing expensive-to-evaluate functions. In our case, we use it to optimize the hyperparameters of the NN architecture. BO allows us to tune hyperparameters in relatively few iterations by building a smooth model from an initial set of parameterizations (referred to as the “surrogate model”) in order to predict the outcomes for as yet unexplored parameterizations. BO builds a smooth surrogate model using Gaussian processes (GPs) based on the observations available from previous rounds of experimentation. This surrogate model is used to make predictions at unobserved parameterizations and quantify the uncertainty around them. The predictions and the uncertainty estimates are combined to derive an acquisition function, which quantifies the value of observing a particular parameterization. We optimize the acquisition function to find the best configuration to observe, and then after observing the outcomes at that configuration a new surrogate model is fitted. This process is repeated until convergence is achieved.

We use the Ax and BoTorch libraries (Facebook, 2019; Balandat et al., 2020; Daulton et al., 2020) to implement the BO based on the expected improvement (EI) acquisition function,

$$\text{EI}\left(x\right)=E[\text{min}\left(f\left(x\right)-f^{\text{*}}\right),0]$$ (7)

where $f^{\text{*}}={\text{min}}_{i}y_{i}$ is the current best observed outcome and our goal is to minimize *f*. The total number of trials is set to 20 with a maximum number of parallel trials of 3 (after the initial exploration). Our target performance metric is the binary cross entropy loss as calculated on a “validation” subset of the jet substructure dataset. After the BO procedure is complete, and a “best” set of hyperparameters is found, each set of hyperparameters tested during the BO procedure is then fully trained for 250 epochs with an early stopping condition, and then metrics are calculated for each model on the “test” subset of the jet substructure dataset.

## 4 Evaluation Metrics

As we develop NN models to address our benchmark application, we use various metrics to evaluate the NNs’ performance. Traditional metrics for performance include the classification accuracy, the receiver operating characteristic (ROC) curve of false positive rate versus true positive rate and the corresponding area under the curve (AUC). In physics applications, it is also important to evaluate the performance in the tails of distributions and we will introduce metrics to measure that as well. The aim of quantization and pruning techniques is to reduce the energy cost of NN implementations, and therefore, we need a metric to measure the computational complexity. For this, we introduce a modified version of BOPs (Baskin et al., 2021). In addition, in this study we aim to understand how the network itself changes during training and optimization based on different NN configurations. While the performance may be similar, we would like to understand if the information is organized in the NN in the same way. Then we would like to understand if that has some effect on robustness of the model. To that end, we explore Shannon entropy metrics (Shannon, 1948) and performance under class randomization.

### 4.1 Classification Performance

**FIGURE 3:**
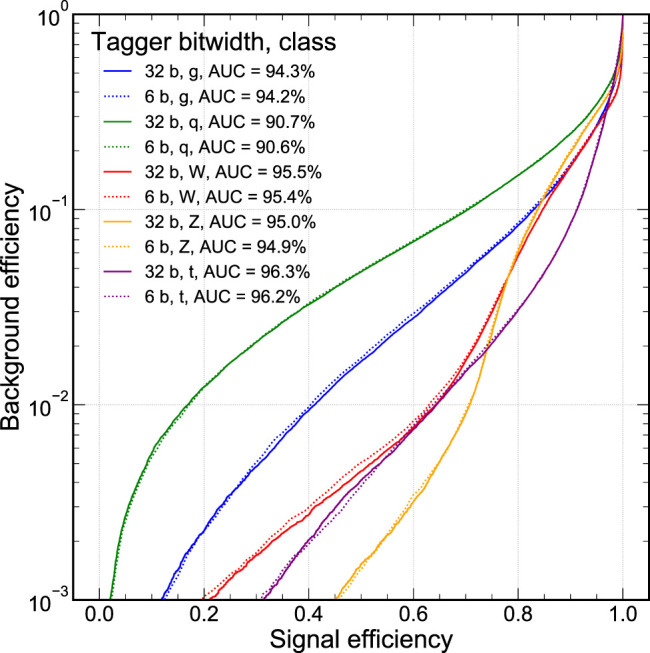
The ROC curve for each signal jet type class where the background are the other four classes. Curves are presented for the unpruned 32-bit floating point classifier (solid lines) and 6-bit scaled integer models (dashed lines). All models are trained with batch normalization layers and $L_{1}$ Regularization.

For our jet substructure classification task, we consider the commonly-used accuracy metric to evaluate for the multi-class performance: average accuracy across the five jet classes. Beyond that, we also want to explore the full shape of the classifier performance in the ROC curve. This is illustrated in Figure 3 where the signal efficiency of each signal class is plotted against the misidentification probability for the other four classes, denoted as the background efficiency. The general features of Figure 3 illustrate that gluon and quark jets are more difficult to distinguish than higher mass jet signals, W and Z boson, and the top quark. The Z boson is typically easier to distinguish than the W boson due to its greater mass. Meanwhile, the top quark is initially the easiest to distinguish at higher signal efficiency but at lower signal efficiencies loses some performance—primarily due to the top quark radiating more because the top quark has color charge. In particle physics applications, it is common to search for rare events so understanding tail performance of a classifier is also important. Therefore, as another performance metric, we define the background efficiency at a fixed signal efficiency of 50%, $\epsilon_{b}^{\epsilon_{s}=0.5}$. We can report this metric $\epsilon_{b}^{\epsilon_{s}=0.5}$ for any signal type, considering all other classes as background processes. From these ROC curves, we see that $\epsilon_{b}^{\epsilon_{s}=0.5}$ can range from a few percent to the per-mille scale for the background samples. In Figure 3, we show the ROC curves for two NN models: one trained with 32-bit floating-point precision and another one trained with QAT at 6-bit scaled-integer precision. The networks are trained with $L_{1}$ regularization and BN layers and do not include pruning.

### 4.2 Bit Operations

The goal of quantization and pruning is to increase the efficiency of the NN implementation in hardware. To estimate the NN computational complexity, we use the BOPs metric (Baskin et al., 2021). This metric is particularly relevant when comparing the performance of mixed precision arithmetic in hardware implementations on FPGAs and ASICs. We modify the BOPs metric to include the effect of unstructured pruning. For a pruned fully-connected layer, we define it as

$$\text{BOPs}=mn\left[\left(1-f_{p}\right)b_{a}b_{w}+b_{a}+b_{w}+{\text{log}}_{2}\left(n\right)\right]$$ (8)

where *n* (*m*) is the number of inputs (outputs), $b_{w}$ ($b_{a}$) is the bit width of the weights (activations), and $f_{p}$ is the fraction of pruned layer weights. The inclusion of the $f_{p}$ term accounts for the reduction in multiplication operations because of pruning. In the dominant term, due to multiplication operations ($b_{a}b_{w}$), BOPs is quadratically dependent on the bit widths and linearly dependent on the pruning fraction. Therefore, reducing the precision is the first step in our QAP procedure, as described above, followed by iterative pruning.

### 4.3 Shannon Entropy, Neural Efficiency, and Generalizability

Typically, the hardware-centric optimization of a NN is a multi-objective, or Pareto, optimization of the algorithm performance (in terms of accuracy or AUC) and the computational cost. Often, we can arrive at a range of Pareto optimal solutions through constrained minimization procedures. However, we would like to further understand how the *information* in different hardware-optimized NN implementations are related. For example, do solutions with similar performance and computational cost contain the same information content? To explore that question, we use a metric called *neural efficiency* $\eta_{N}$ (Schaub and Hotaling, 2020).

Neural efficiency measures the utilization of state space, and it can be thought of as an entropic efficiency. If all possible states are recorded for data fed into the network, then the probability, $p_{s}$, of a state *s* occurring can be used to calculate Shannon entropy $E_{\ell }$ of network layer ℓ

$$E_{\ell }=-\overset{S}{\underset{s=1}{\sum }}p_{s}{\text{log}}_{2}\left(p_{s}\right),$$ (9)

where the sum runs over the total size of the state space *S*. For a *b*-bit implementation of a network layer with $N_{\ell }$ neurons, this sum is typically intractable to compute, except for extremely low bit precision and small layer size, as the state space size is $S=2^{bN_{\ell }}$ Therefore, a simplification is made to treat the state of a single neuron as binary (whether the output value is greater than zero) so that $S=2^{N_{\ell }}$. The maximum entropy of a layer corresponds to the case when all states occur with equal probability, and the entropy value is equal to the number of neurons $E_{\ell }=N_{\ell }$. The neural efficiency of a layer can then be defined as the entropy of the observed states relative to the maximum entropy: $\eta_{\ell }=E_{\ell }/N_{\ell }$. Neuron layers with neural efficiency close to one (zero) are making maximal (minimal) usage of the available state space. Alternatively, high neural efficiency could also mean the layer contains too few neurons.

To compute the neural efficiency of a fully-connected NN $\eta_{N}$ we take the geometric mean of the neural efficiency of each layer $\eta_{\ell }$ in the network

$$\eta_{N}={\left(\overset{L}{\underset{\ell =1}{\prod }}\eta_{\ell }\right)}^{\frac{1}{L}}$$ (10)

Although neural efficiency $\eta_{N}$ does not directly correlate with NN performance, in Schaub and Hotaling (2020), it was found there was connection between NN generalizability and the neural efficiency. NNs with higher neural efficiency that maintain good accuracy performance were able to perform better when classes were partially randomized during training. The interpretation is that such networks were able to learn general features of the data rather than memorize images and therefore are less susceptible to performance degradation under class randomization. Therefore, in the results of our study, we also explore the effect of class randomization on our jet substructure task.

## 5 Results

In the previous sections, we have introduced the benchmark task, the QAP approach, and metrics by which we will evaluate the procedure. In this section, we present the results of our experiments. Our experiments are designed to address three conceptual topics:

• In Section 5.1, we aim to study how certain training configuration choices can affect the performance (accuracy and $\epsilon_{b}^{\epsilon_{s}=0.5}$) of our QAP procedure and how it compares to previous works. In particular, we study the dependence of performance on the pruning procedure, the bit width, and whether we include batch normalization and $L_{1}$ regularization into the network training.
• In Section 5.2, now with an optimized procedure for QAP, we would like to understand the relationship between structured (neuron-wise) and unstructured (synapse-wise) pruning. These two concepts are often overloaded but reduce computational complexity in different ways. To do this, we compare the unstructured pruning procedure we introduced in Section 5.1 to removing whole neurons in the network. Structured pruning, or optimizing the hyperparameter choice of NN nodes, is performed using a Bayesian Optimization approach introduced in Section 3.3.2.
• In Section 5.3, we make preliminary explorations to understand the extent to which QAP is removing important synapses which may prevent generalizability of the model. While there are a number of ways to test this; in our case, we test generalizability by randomizing a fraction of the class labels and checking if we are still able to prune the same amount of weights from the network as in the non-randomized case.

### 5.1 Quantization—Aware Pruning Performance

**FIGURE 4:**
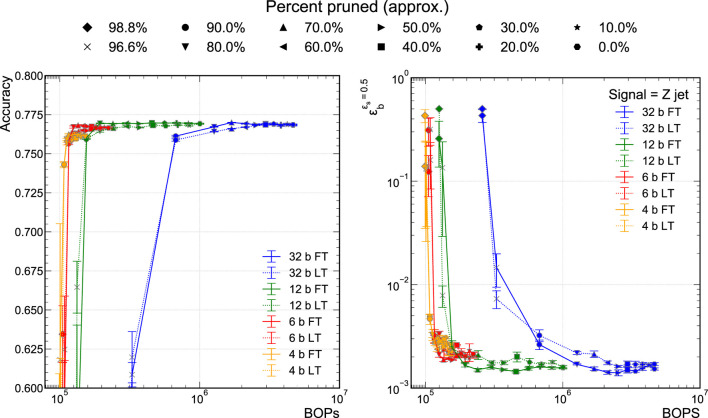
Model accuracy **(A)** and background efficiency **(B)** at 50% signal efficiency versus BOPs for different sparsities achieved *via* QAP, for both FT and LT pruning techniques.

The physics classifier performance is measured with the accuracy and $\epsilon_{b}^{\epsilon_{s}=0.5}$ metric for each signal class. We train a number of models at different precision: 32-bit floating-point precision and 12-, 6-, and 4-bit scaled-integer precision. For each precision explored, we then apply a pruning procedure. We explore both of the LT and FT pruning schemes described in Section 3. The result is illustrated in Figure 4 where each of the colored lines indicates a different model precision, the solid (dashed) lines correspond to FT (LT) pruning, and each of the points along the curves represents the percent of the original network weights that have been pruned. Each NN includes a BN layer after each of the hidden layers and has been trained including an $L_{1}$ regularization loss term. Further, each model’s performance was verified *via* a *k*-fold cross-validation scheme, where k=4 in which training and validation datasets were shuffled over multiple training instances. Plotted performance is the mean value and error bars represent the standard error across the folds. All metrics were calculated on the same test dataset, which stayed static across each training instance. The first observation from Figure 4 is that we can achieve comparable performance to the 32-bit floating-point model with the 6-bit scaled-integer model. This is consistent with findings in a previous QKeras-based study (Coelho et al., 2021) where, with uniform quantization, the performance was consistent down to 6-bit fixed-point quantization. When the precision is reduced to 4-bits, the performance begins to degrade. Then, as we increasingly prune the models at all of the explored precisions, the performance is maintained until about 80% of the weights are pruned. The observations are consistent whether we consider the accuracy (Figure 4A) or $\epsilon_{b}^{\epsilon_{s}=0.5}$ (Figure 4B right) metric. For the case of $\epsilon_{b}$, there is an increase of roughly 1.2–2 × with respect to the 32-bit floating-point model; however, there are statistical fluctuations in the values because of the limited testing sample size and the small background efficiencies of $2\times 10^{-3}$ that we probe. Instead, now if we compare the computational cost of our QAP 6-bit model to the unpruned 32-bit model, we find a greater than 25× reduction in computational cost (in terms of BOPs) for the same classifier performance. For the jet substructure classification task, the quantization and pruning techniques are complementary and can be used in tandem at training time to develop an extremely efficient NN. With respect to earlier work with FT pruning at 32-bit floating-point precision and PTQ presented in Duarte et al. (2018), we find a further greater than 3× reduction in BOPs.

In Figure 4, we also find that there is no significant performance difference between using FT and LT pruning. As we prune the networks to extreme sparsity, greater than 80%, the performance begin to degrade drastically for this particular dataset and network architecture. While the plateau region is fairly stable, in the ultra-sparse region, there are significant variations in the performance metrics indicating that the trained networks are somewhat brittle. For this reason, we truncate the accuracy versus BOPs graphs at 60% accuracy.

**FIGURE 5:**
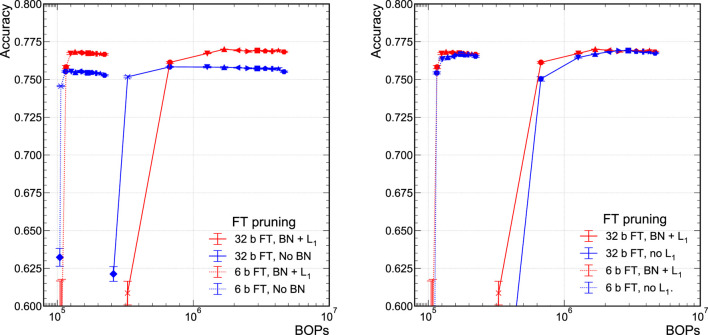
Comparison of the model accuracy when trained with BN layers and $L_{1}$ regularization versus when trained without BN layers **(A)** or $L_{1}$ regularization **(B)**.

We also explore the performance of the model when removing either the BN layers or the $L_{1}$ regularization term, which we term the *no BN* and *no* $L_{1}$ models, respectively. This is illustrated in Figure 5 for the 32-bit floating-point and 6-bit scaled-integer models. For easier visual comparisons, we omit the 4-bit and 12-bit models because the 6-bit model is the lowest precision model with comparable performance to the 32-bit model. In Figure 5A, we see that there is a modest performance degradation in the no BN configuration for both lower and full precision models. In our application, we find that batch normalization does stabilize and improve the performance of our NN and thus include it in our baseline model definition. In Figure 5B, we find that including or removing the $L_{1}$ regularization term in the loss function does not affect the performance significantly until extreme sparsity where the variations in performance can be large. However, as we will see in Section 5.3, this does not mean that the entropic information content of the NNs are similar.

To highlight the performance of the QAP procedure, we summarize our result compared to previous results for this jet substructure classification task with the same NN architecture shown in Figure 1. The results are summarized in Table 1. In the nominal implementation, no quantization or pruning is performed. In Duarte et al. (2018), the 32-big floating-point model is FT pruned and then quantized post-training. This approach suffers from a loss of performance below 16 bits. Using QAT and QKeras (Coelho et al., 2021), another significant improvement was demonstrated with a 6-bit fixed-point implementation. Finally, in this work with QAP and Brevitas, we are able to prune the 6-bit network by another 80%. With respect to the nominal implementation we have reduced the BOPs by a factor of 25, the original pruning + PTQ approach a factor of 3.3, and the QAT approach by a factor of 2.2.

One further optimization step is to compare against a mixed-precision approach where different layers have different precisions (Coelho et al., 2021). We leave the study of mixed-precision QAP to future work and discuss it in Section 6.

### 5.2 Pruned Versus Unpruned Quantized Networks

To compare against the efficacy of applying QAP, we explore QAT with no pruning. In an alternate training strategy, we attempt to optimize the NN architecture of the unpruned QAT models. This is done using the BO technique presented in Section 3.3. The widths of the hidden layers are varied to find optimal classifier performance. We compare the performance of this class of possible models using BO against our QAP procedure, including BN and $L_{1}$ regularization, presented in the previous section. It is important to note, as we will see, that QAP and BO are conceptually different procedures and interesting to compare. The QAP procedure starts with a particular accuracy-optimized model and attempts to “streamline” or compress it to its most optimal bit-level implementation. This is the reason that the accuracy drops precipitously when that particular model can no longer be streamlined. Alternatively, the family of BO models explores the Pareto optimal space between BOPs and accuracy. In future work, we would like to further explore the interplay between QAP and BO.

**FIGURE 6:**
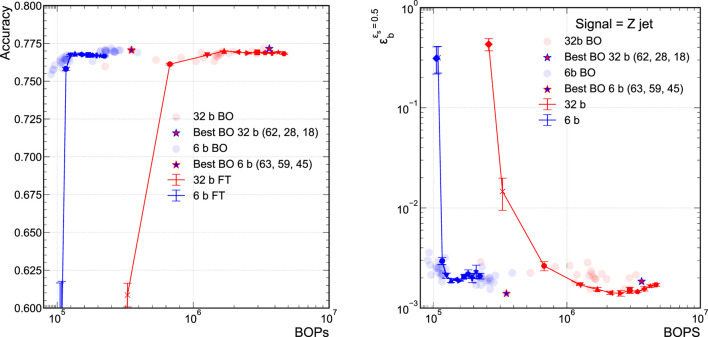
Comparison of FT pruned model’s and BO model’s accuracy **(A)** and background efficiency (**B**) at 50% signal efficiency. Each hyperparameter configuration that was explored during the BO procedure is marked as a transparent dot, with the resulting “best” model, which the lowest BCE Loss as calculated on the “test” set, is marked by the outlined star.

Figure 6 presents both the accuracy versus BOPs curves for the QAP models and the unpruned QAT models found using BO. For ease of comparison, we display only the 32-bit and 6-bit models. The solid curves correspond to the QAP models while the individual points represent the various trained unpruned models explored during the BO procedure. The unpruned model with the highest classification performance found using the BO procedure is denoted by the star. While the starred models are the most performant, there is a class of BO models that tracks along the QAP curves fairly well. There is a stark difference in how QAP and BO models behave as the accuracy degrades below the so-called “plateau” region where the accuracy is fairly constant and optimal. When the sub-network of the QAP model can no longer approximate the optimally performing model, its performance falls off dramatically and the accuracy drops quickly. Because BO explores the full space including Pareto optimal models in BOPs versus accuracy, they exhibit a more gentle decline in performance at small values of BOPs. It is interesting to note that the classification performance of the BO models begins to degrade where the QAP procedure also falls off in performance; for example, just above $10^{5}$/BOPs in Figure 6A for the 6-bit models. We anticipate future work to explore combining BO and QAP procedures to see if any accuracy optimal model can be found at smaller BOPs values.

### 5.3 Entropy and Generalization

QAP models exhibit large gains in computational efficiency over (pruned and unpruned) 32-bit floating-point models, as well as significant gains over unpruned QAT models for our jet substructure classification task. In certain training configurations, we have found similar performance but would like to explore if the information in the NN is *represented* similarly. As a metric for the information content of the NN, we use the *neural efficiency* metric defined in Eq. 10, the Shannon entropy normalized to the number of neurons in a layer then averaged over all the layers of the NN.

By itself, the neural efficiency is an interesting quantity to measure. However, we specifically explore the hypothesis, described in Section 4, that the neural efficiency is related to a measure of generalizability. In this study, we use the classification performance under different rates of class randomization during training as a probe of the generalizability of a model. We randomize the class labels among the five possible classes for 0, 50, 75, and 90% of the training dataset. To randomize the training data, we iterate over a given percent of the normal dataset, setting the real class of each input to 0, choosing a new class at random out of the 5 possible, then setting that new class to 1. The data is then shuffled and split as normal.

**FIGURE 7:**
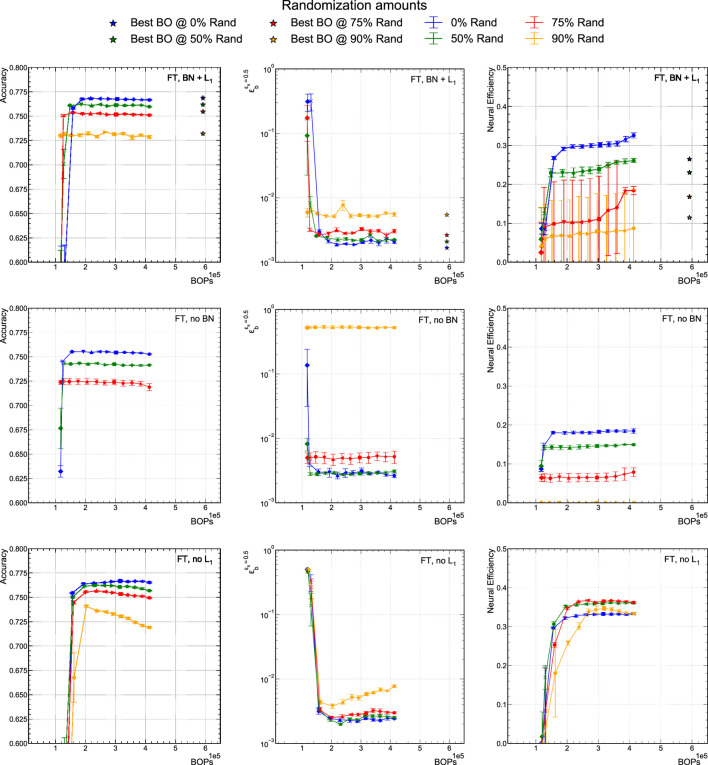
Comparison of accuracy, $\epsilon_{b}^{\epsilon_{s}=0.5}$, and neural efficiency at 50% signal efficiency for a 6-bit QAP model as BN layers and/or $L_{1}$ regularization is present in the model. $L_{1}$ + BN **(upper)**, no BN **(middle)**, and no $L_{1}$ **(lower)**.

To compare with the results in Section 5.1, we study models that are trained using QAP with 6-bit precision and are pruned using the fine-tuning pruning procedure. The results are presented in Figure 7 where the left column shows the classifier accuracy versus BOPs. The center column shows the $\epsilon_{b}^{\epsilon_{s}=0.5}$ metric. The right column displays the neural efficiency versus BOPs. The three rows explore three different scenarios: with both BN and $L_{1}$ regularization (upper), no BN (middle), and no $L_{1}$ (lower). The various curves presented in each graph correspond to different class label randomization fractions of the training sample.

Among these training procedures, the $L_{1}$+ BN model accuracy (upper left) is the highest and most consistent across the entire pruning procedure. Even with 90% class randomization, the accuracy is still greater than 72.5% and $\epsilon_{b}^{\epsilon_{s}=0.5}<10^{-2}$. Alternatively, the no BN model accuracy is consistently worse than the $L_{1}$ + BN models for all values of randomization. Interestingly, the no BN model accuracy with 90% randomization drops precipitously out of the range of the graphs indicating that BN is even more important to performance when class randomization is introduced. Meanwhile, the no $L_{1}$ model exhibits an interesting behavior with lower accuracy at larger values of BOPs. As the no $L_{1}$ model is pruned, the accuracy improves until we arrive at extreme sparsity and the model performance degrades as usual. Our interpretation is that the generalization power of the unregularized model is worse than the $L_{1}$ regularized models. However, as we implement the QAP procedure, the pruning effectively regularizes the model building robustness to the class randomization and recovering some of the lost accuracy.

The corresponding neural efficiency plots are shown in the right column of Figure 7. As a general observation, we find that the neural efficiency follows the same trend versus BOPs as the accuracy, i.e., that within a given training configuration, the neural efficiency is stable up to a given sparsity. Thus, up to this point, pruning does not affect the information content. This is particularly true in the case of the no BN model, while with BN there is more freedom, and thus modest variation in neural efficiency during the pruning procedure.

If we first only consider the 0% randomized models for the right column, we can see that the neural efficiency drops from about 0.3 to about 0.2 with the no BN configuration. As the neural efficiency is a measure of how balanced the neurons are activated (i.e., how efficiently the full state space is used), we hypothesize that BN more evenly distributes the activation among neurons. For the models that include $L_{1}$ regularization (upper and middle), the neural efficiency drops along with the accuracy as the randomization is increased. This effect is not nearly as strong in the no $L_{1}$ case in the lower row. We note that the performance of the 90% randomized no BN model is catastrophically degraded and the neural efficiency drops to zero, which we interpret to indicate that BN is an important factor in the robustness and generalizability of the model.

The no $L_{1}$ models (lower) are particularly notable because the neural efficiency does not decrease much as we the class randomization fraction is increased, in contrast with the upper and middle rows of Figure 7. This however, does not translate into a more robust performance. In fact, at 90% class randomization and 80% pruned, the *L*_1_ + BN and no *L*_1_ models are drastically different in neural efficiency while being fairly similar in classifier accuracy.

Finally, the accuracy and neural efficiency of the highest accuracy models from the BO procedure in Section 5.2 are represented as stars in the top row of Figure 7. They have slightly lower neural efficiencies because the width of each hidden layer is bigger than in the QAP models while the entropy remains relatively similar to those same models. The BO models, as seen in the upper left graph of Figure 7, are no better at generalizing under increasing class randomization fractions than the QAP models.

## 6 Summary and Outlook

In this study, we explored efficient NN implementations by coupling pruning and quantization at training time. Our benchmark task is ultra low latency, resource-constrained jet classification in the real-time online filtering system, implemented on field-programmable gate arrays (FPGAs), at the CERN Large Hadron Collider (LHC). This classification task takes as inputs high-level expert features in a fully-connected NN architecture.

Our procedure, called QAP, is a combination of QAT followed by iterative unstructured pruning. This sequence is motivated by the fact that quantization has a larger impact on a model’s computational complexity than pruning as measured by BOPs. We studied two types of pruning: fine-tuning (FT) and lottery ticket (LT) approaches. Furthermore, we study the effect of batch normalization (BN) layers and $L_{1}$ regularization on network performance. Under this procedure, considering networks with uniformly quantized weights, we found that with nearly no loss in classifier accuracy and 1.2−2× increase in $\epsilon_{b}$, the number of BOPs can be reduced by a factor of 25, 3.3, and 2.2 with respect to the nominal 32-bit floating-point implementation, pruning with post-training quantization (PTQ), and QAT, respectively. This demonstrates that, for our task, pruning and QAT are complementary and can be used in concert.

Beyond computational performance gains, we sought to understand two related issues to the QAP procedure. First, we compare QAP to QAT with a Bayesian optimization (BO) procedure that optimizes the layer widths in the network. We found that the BO procedure did not find a network configuration that maintains performance accuracy with fewer BOPs and that both procedures find similarly efficiently sized networks as measured in BOPs and high accuracy.

Second, we studied the information content, robustness, and generalizability of the trained QAP models in various training configurations and in the presence of randomized class labels. We compute both the networks’ accuracies and their entropic information content, measured by the neural efficiency metric (Schaub and Hotaling, 2020). We found that both $L_{1}$ regularization and BN are required to provide the most robust NNs to class randomization. Interestingly, while removing $L_{1}$ regularization did not significantly degrade performance under class randomization, the neural efficiencies of the NNs were vastly different—varying by up to a factor of 3. This illustrates, that while NNs may arrive at a similar performance accuracy, the information content in the networks can be very different.

### 6.1 Outlook

As one of the first explorations of pruning coupled with quantization, our initial study of QAP lends itself to a number of follow-up studies.

• Our benchmark task uses high-level features, but it is interesting to explore other canonical datasets, especially those with raw, low-level features. This may yield different results, especially in the study of generalizability.
• Combining our approach with other optimization methods such as Hessian-based quantization (Dong et al., 2019; Dong et al., 2020) and pruning could produce networks with very different NNs in information content or more optimal solutions, particularly as the networks become very sparse.
• An important next step is evaluating the actual hardware resource usage and latency of the QAP NNs by using FPGA co-design frameworks like hls4ml (Duarte et al., 2018) and FINN (Umuroglu et al., 2017; Blott et al., 2018).
• It would be interesting to explore the differences between seemingly similar NNs beyond neural efficiency; for example, using metrics like singular vector canonical correlation analysis (SVCCA) (Raghu et al., 2017) which directly compare two NNs
• We would like to explore further optimal solutions by combining BO and QAP procedures. Beyond that, there is potential for more efficient solutions using mixed-precision QAT, which could be done through a more general BO procedure that explores the full space of layer-by-layer pruning fractions, quantization, and sizes.

QAP is a promising technique to build efficient NN implementations and would benefit from further study on additional benchmark tasks. Future investigation of QAP, variations on the procedure, and combination with complementary methods may lead to even greater NN efficiency gains and may provide insights into what the NN is learning.

## Data Availability

Publicly available datasets were analyzed in this study. This data can be found here: https://zenodo.org/record/3602254.
